# Elevated high-sensitivity C-reactive protein levels increase the risk of new-onset cardiac conduction disorders

**DOI:** 10.1186/s12933-023-01987-1

**Published:** 2023-09-30

**Authors:** Lili Wu, Meimei Wu, Dandan Zhao, Shuohua Chen, Guodong Wang, Lina Xu, Yujing Wang, Lina An, Shouling Wu, Congliang Miao, Jiang Hong

**Affiliations:** 1 Department of Cardiology, Shanghai Songjiang District Central Hospital, Shanghai, China; 2 Department of Emergency and Critical Care Medicine, Shanghai Songjiang District Central Hospital, Shanghai, China; 3 Division of Cardiovascular Diseases, Department of Emergency and Critical Care Medicine, Shanghai General Hospital, Shanghai Jiao Tong University School of Medicine, No.100 Haining Road, Hongkou District, Shanghai, 200080 China; 4 Department of Cardiology, Kailuan Hospital, North China University of Science and Technology, No. 57 Xinhua East Road, Tangshan, 063001 China

**Keywords:** Inflammation, Conduction disorders, High-sensitivity C-reactive protein, Atrioventricular block, Bundle branch block

## Abstract

**Background:**

Previous studies have reported that inflammatory responses can promote the onset of cardiovascular diseases; however, its association with cardiac conduction disorders remains unclear. The present community-based cohort study aimed to elucidate the effects of inflammatory responses on the risk of developing cardiac conduction disorders.

**Methods:**

After the exclusion of participants failing to meet the inclusion criteria, 86,234 eligible participants (mean age: 50.57 ± 11.88 years) were included. The participants were divided into high-sensitivity C-reactive protein (hsCRP)  ≤  3 mg/L, and hsCRP > 3 mg/L groups based on hsCRP values. Multivariate Cox proportional hazard model was used to analyze the relationship between inflammatory responses and various cardiac conduction disorders.

**Results:**

After adjusting for confounding factors, we observed that compared with the hsCRP ≤ 3 mg/L group, the hsCRP > 3 mg/L group exhibited increased risks of atrioventricular block (hazard ratio [HR]:1.64, 95%confidence interval [CI] 1.44–1.87) and left (HR:1.25, 95% CI 1.07–1.45) and right bundle branch block (HR:1.31, 95% CI 1.17–1.47). Moreover, the risk of various cardiac conduction disorders increased for every 1 standard deviation increase in log (hsCRP). The restricted cubic spline function confirmed a linear relationship between log (hsCRP) and the risk of developing cardiac conduction disorders (All nonlinearity *P* > 0.05).

**Conclusions:**

High hsCRP levels are an independent risk factor for cardiac conduction disorders, and hsCRP levels are dose-dependently associated with the risk of conduction disorders. Our study results may provide new strategies for preventing cardiac conduction disorders.

**Supplementary Information:**

The online version contains supplementary material available at 10.1186/s12933-023-01987-1.

## Introduction

Cardiac conduction disease is a condition characterized by permanent or temporary disruptions in the transmission of electrical impulses within the cardiac conduction system. These disruptions can result from structural or functional abnormalities occurring at various levels of the cardiac conduction system. Notably, it often presents as bradycardia and is quite prevalent. Recent data from different European countries has revealed that prolonged PR intervals are observed in 1.9% to 3.7% of cases [1–3]. In China, in the year 2018, the prevalence of atrioventricular blocks was approximately 7.06% [4]. Previous studies have established a link between cardiac conduction disorders and an elevated risk of adverse cardiac outcomes. For instance, findings from the Framingham Heart Study indicate that a prolonged PR interval (> 200 ms) is associated with a 1.44-fold increase in the risk of all-cause mortality [5]. Furthermore, the Cardiovascular Health Study has reported that a left anterior branch block is linked to a 2.43-fold increased risk of heart failure and a 1.57-fold increased risk of all-cause mortality [6]. Despite the evident association between cardiac conduction disorders and poor prognosis, research on the risk factors and prognosis of these disorders is limited. Our investigation of the PubMed database, spanning from 2000 to 2023, uncovered fewer than 10 prospective cohort studies focusing on the risk factors and prognosis of this condition.

Although a few studies have identified the risk factors for cardiac conduction disorders, including advanced age, male gender, hypertension, and diabetes [7–11], they do not comprehensively explain the risk of their development. Myocardial fibrosis is thought to be an important pathological substrate for the development of cardiac conduction disorders [12, 13], which can interfere with all the basic electrophysiological mechanisms leading to arrhythmogenesis, including delayed action potential propagation, afterdepolarizations, re-entry, and increased ectopic automaticity [14]. Furthermore, inflammatory responses can promote the formation and development of myocardial fibrosis via electrical and structural remodeling [13, 15]. However, to the best of our knowledge, very few cohort studies have demonstrated if inflammatory responses can increase the risk of developing conduction disorders. Recently, Emilie et al. [16] reported that elevated high-sensitivity C-reactive protein (hsCRP) levels were associated with an increased risk of developing cardiac conduction disorders; however, their observations were in an elderly population with a small cohort size. Therefore, whether inflammation can increase the risk of developing conduction disorders in the general population remains unelucidated, which may provide a new possible direction of future strategies for preventing cardiac conduction disorders. The present study aimed to determine the effects of inflammatory responses on the risk of developing cardiac conduction disorders using data from the Kailuan study.

## Subjects and methods

### Study population

In this prospective cohort study, a total of 11 hospitals in Kailuan General Hospital and affiliated hospitals conducted health check-ups for their active and retired employees from 2006, followed by follow-ups every 2 years; follow-up experiments included hsCRP measurement and a routine electrocardiography (ECG) examination. We enrolled Kailuan workers who participated in the 2006 health check-up as the participants. The inclusion criteria were as follows: (1) participants who attended the 2006 health check-up; (2) those with complete hsCRP and ECG data; and (3) those who agreed to participate in the study and signed the informed consent form. The exclusion criteria were as follows: (1) participants who attended the 2006 health check-up but did not participate in the follow-up check-ups; (2) those who were diagnosed with cardiac conduction disorders at the 2006 medical check-up; (3) those with arrhythmias such as atrial fibrillation, pre-excitation syndrome, and ventricular tachycardia or those with permanent pacemaker implantation; individuals with a previous diagnosis of congestive heart failure; and (5) individuals who have used beta-blockers and nondihydropyridine calcium channel blockers. This study has been approved by the Ethics Committee of Kailuan General Hospital in accordance with the Helsinki Declaration.

### Data collection

#### Collection of general clinical characteristics, laboratory tests, and related definitions

Information on the age, sex, disease status, and medication use of the study participants was collected using questionnaires. The methods and criteria for determining height, weight, and relevant biochemical indicators were according to a previous study [17]. Further, information on the diagnosis of new-onset myocardial infarction was collected via discharge records from all 11 hospitals in Kailuan, covering all participants in the Kailuan study. Smoking was defined as an average of at least 1 cigarette per day for the last 1 year, and for those who had quit smoking for < 1 year. Alcohol consumption was defined as an average of 100 mL of white wine (≥ 50% alcohol) per day for the last 1 year, and for those who have abstained from drinking for < 1 year. Physical activity was defined as exercise for ≥ 3 times per week for ≥ 30 min. Body mass index (BMI) was calculated using the following formula: BMI = weight/height^2^ (kg/m^2^). Estimated glomerular filtration rate (eGFR) was calculated using the Chronic Kidney Disease Epidemiology Collaboration formula [18]. Hypertension was defined as a systolic blood pressure (SBP) of ≥ 140 mm Hg and/or diastolic blood pressure (DBP) of ≥ 90 mmHg or the use of antihypertensive medication or history of hypertension despite a BP of < 140/90 mmHg [19]. Diabetes mellitus was defined as fasting blood glucose (FBG) levels of ≥ 7 or < 7 mmol/L but with a previous diagnosis of diabetes mellitus or those currently being treated with glucose-lowering drugs [20].

#### Measurement of hsCRP levels and grouping

The participants fasted for at least 8 h. On the day of the physical examination, 5 mL of fasting elbow venous blood was collected into EDTA vacuum tubes for the assay at 7:00–9:00 am. The plasma was separated and stored at − 80 °C for further analysis. HsCRP levels were determined using a high-sensitivity enhanced immunoturbidimetric assay (Cias Latex CRP-H. Kanto Chemical Co. Inc., Tokyo, Japan), with intra- and interassay coefficients of variation of 6.53% and 4.78%, respectively, and a lower limit of detection of 0.1 mg/L. All plasma samples were tested at the central laboratory of Kailuan General Hospital using an automated analyzer (Hitachi 747, Hitachi, Tokyo. Japan).

As per the guideline [21], high hsCRP levels were defined as serum hsCRP levels of > 3 mg/L; the study population was accordingly divided into the hsCRP ≤ 3 mg/L group, and the hsCRP > 3 mg/L group.

#### ECG measurements and definition of endpoint events

Routine ECG measurements were performed at the 2006 health check-up and subsequent follow-up visits every 2 years. Subjects comfortably rested in the supine position in a quiet room for 5 min before a 12-lead ECG was taken for 10 s from 7:00–9:00 am. ECG measurements and diagnosis were completed by two ECG specialists. Various cardiac conduction disorders were defined according to the ECG diagnostic criteria of the Minnesota Code and the relevant US AHA guidelines [22–24], as detailed in Additional file 1: Table S1.

The primary endpoint was defined as any cardiac conduction disorder (aCCD), including I–III atrioventricular block (AVB), complete right bundle branch block (CRBBB), incomplete right bundle branch block (iRBBB), complete left bundle branch block (CLBBB), incomplete Left bundle branch block (iLBBB), left anterior fascicular block (LAFB), left posterior fascicular block (LPFB), and nonspecific intraventricular conduction disturbance (NS-IVCD). On the other hand, when defining each specific conduction disorder as an endpoint event, any AVB (aAVB) was defined as I, II, and III AVB; any bundle branch block (aBBB) was defined as CRBBB, iRBBB, CLBBB, iLBBB, LAFB, and LPFB; any left bundle branch block (aLBBB) was defined as CLBBB, iLBBB, LAFB, and LPFB; and right bundle branch block (RBBB) was defined as CRBBB and iRBBB [22–24].

### Follow-up of endpoint events

The date of completion of the 2006 health check-up was used as the starting point for follow-up experiments, and the first new cardiac conduction block was used as the endpoint event, with the time of occurrence as the endpoint time. For participants without any cardiac conduction disorders, the endpoint time was the date of the last follow-up visit. The occurrence of the endpoint event was recorded after reviewing the ECG follow-up results every 2 years for a total of six follow-up visits between 2008 and 2018, with a follow-up cutoff date of December 31, 2019.

### Statistical analysis

Normally distributed data were expressed as mean ± standard deviation. Two-group comparisons were performed using the two independent samples t-test, whereas multigroup comparisons were performed using a one-way analysis of variance. Nonnormally distributed data were expressed as median and quartile spacing (P25–P75). The Kruskal–Wallis rank sum test was used for between-group comparisons. Counting data were expressed as frequencies and percentages (n, %), and between-group comparisons were performed using the chi-squared test. The study population was divided into two groups based on whether the hsCRP level was > or < 3 mg/L. The cumulative incidence of cardiac conduction disorder events in different groups was calculated using the Kaplan–Meier method and compared using the log-rank test. Multifactorial Cox proportional risk regression models were used to estimate hazard ratios (HRs) between different hsCRP subgroups or the HRs per one standard deviation increase in log (hsCRP) and 95% confidence intervals (CIs), and the age, sex, BMI, physical activity, smoking status, alcohol consumption, cholesterol (TC), uric acid (UA), eGFR at baseline, hypertension, diabetes mellitus, myocardial infarction at baseline or during follow-up, and uses of antihypertensive, lipid-lowering, and glucose-lowering drugs were corrected at baseline or during follow-up. The relationship between log (hsCRP) as a continuous variable and the risk of various conduction disorders was determined using restrictive triplicate strips after correcting for potential confounders. Subgroup analyses were conducted independently for age and gender to further pinpoint specific populations of interest. Sensitivity analysis was undertaken to validate the findings by reclassifying participants with or without hsCRP ≥ 1 mg/L, by excluding those with hsCRP levels > 10 mg/L, and by excluding individuals with a baseline MI history. This approach allowed us to once again compare the risk of developing cardiac conduction disorders between these two groups. Considering the long follow-up period of this study, a time-dependent Cox proportional risk regression model was established for the overall population to determine the effects of short-time exposure on the risk of developing cardiac conduction disorders. Furthermore, a competing risk model of death was established for the overall population, considering the effects of death on cardiac conduction disorder events during the follow-up. All data were analyzed using SAS statistical software (Version 9.4; SAS Institute, Cary, NC). Differences were considered statistically significant at a *P*-value of < 0.05 (two-sided test).

## Results

**Fig. 1:**
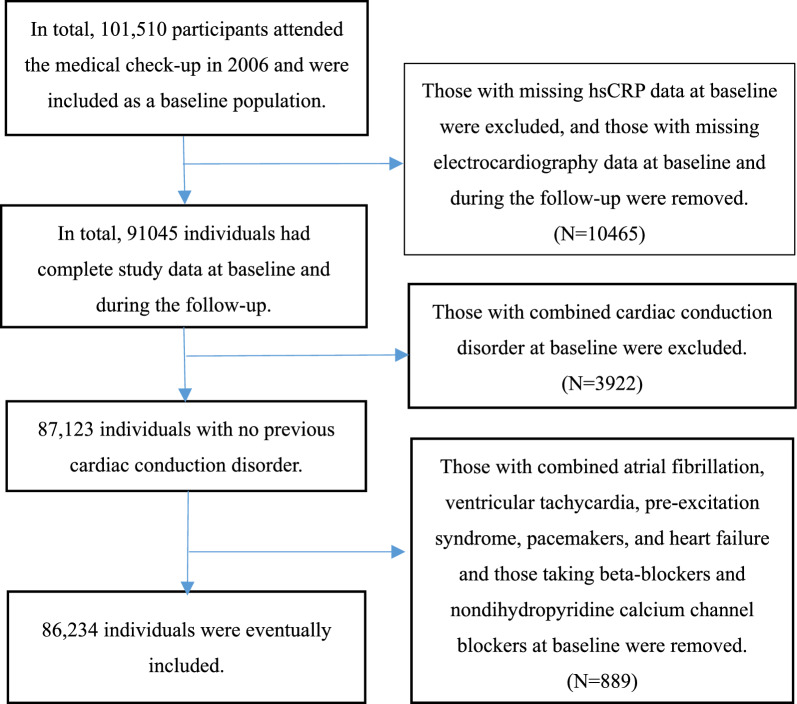
Study flow chart

In total, 101,510 individuals participated in the medical check-up in 2006. Among these individuals, 3768 were excluded due to incomplete high-sensitivity hsCRP data at baseline, 6697 were excluded due to incomplete ECG data at both baseline and during the follow-up period, 3922 were excluded due to the presence of concurrent cardiac conduction disorders at baseline, and 889 were excluded due to the presence of combined conditions, including atrial fibrillation, ventricular tachycardia, pre-excitation syndrome, permanent pacemaker implantation, congestive heart failure, as well as the use of beta-blockers and nondihydropyridine calcium channel blockers at baseline. Finally, 86,234 individuals were included in the statistical analysis (Fig. 1).

### Baseline characteristics

The mean age of the participants was 50.57 ± 11.88 years; 67,665 (78.47%) participants were men. The mean SBP was 130.23 ± 20.62 mmHg, and the median hsCRP level was 0.80 (0.30–2.11) mg/L. A total of 70,002 (81.17%) participants were in the hsCRP ≤ 3 mg/L group and 16,232 (18.83%) participants were in the hsCRP > 3 mg/L group. Age, BMI, TC, eGFR, FBG, SBP, DBP, number of heart attacks at baseline, proportion of participants with hypertension at baseline, proportion of participants with diabetes mellitus at baseline, and proportion of participants with taking antihypertensive, lipid-lowering, and glucose-lowering drugs at baseline were higher in the hsCRP > 3 mg/L group than in the hsCRP ≤ 3 mg/L group (*P* < 0.05). The percentages of males, smoking, alcohol consumption and physical exercise in the hsCRP > 3 mg/L group were lower than those in the hsCRP ≤ 3 mg/L group (*P* < 0.05); no statistical difference in uric acid levels between the two groups (*P* > 0.05), see Table 1.

**Table 1:** Baseline and the follow-up characteristics of the study participants

	Overall	hsCRP ≤ 3 mg/L	hsCRP > 3 mg/L	*P* value
Participant (%)	86,234 (100)	70,002 (81.17)	16,232 (18.83)	
Age (year)	50.57 ± 11.88	49.76 ± 11.72	54.13 ± 11.93	< 0.0001
Male [n (%)]	67,665 (78.47)	55,317 (79.02)	12,348 (76.07)	< 0.0001
BMI (kg/m^2^)	25.07 ± 3.46	24.93 ± 3.38	25.66 ± 3.71	< 0.0001
TC (mmol/L)	4.95 ± 1.14	4.95 ± 1.14	4.97 ± 1.11	0.045
UA (umol/L)	287.08 ± 82.65	287.04 ± 80.74	287.28 ± 90.42	0.735
eGFR (ml/min/1.73m^2^)	72.32 ± 25.25	71.85 ± 24.58	74.34 ± 27.85	< 0.0001
hsCRP (mg/L)	0.80 (0.30 ~ 2.11)	0.58 (0.23 ~ 1.19)	6.30 (4.12 ~ 9.20)	< 0.0001
FBG (mmol/L)	5.45 ± 1.62	5.42 ± 1.52	5.57 ± 1.94	< 0.0001
SBP (mmHg)	130.23 ± 20.62	129.56 ± 20.31	133.10 ± 21.68	< 0.0001
DBP (mmHg)	83.38 ± 11.67	83.19 ± 11.58	84.21 ± 11.98	< 0.0001
Smoking [n (%)]	33,835 (39.24)	28,490 (40.70)	5345 (32.93)	< 0.0001
Drinking [n (%)]	34,921 (40.50)	29,577 (42.25)	5344 (32.92)	< 0.0001
Physical activity [n (%)]	76,734 (88.98)	63,306 (90.43)	13,428 (82.73)	< 0.0001
Proportions of relevant diseases or taking relevant medications at baseline				
Myocardial Infarction [n (%)]	965 (1.12)	732 (1.05)	233 (1.44)	< 0.0001
Hypertension [n (%)]	36,992 (42.90)	28,869 (41.24)	8123 (50.04)	< 0.0001
Diabetes [n (%)]	7659 (8.88)	5708 (8.15)	1951 (12.02)	< 0.0001
Antihypertensive drugs [n (%)]	8891 (10.31)	6820 (9.74)	2071 (12.76)	< 0.0001
Hypoglycemic drugs [n (%)]	1915 (2.22)	1467 (2.10)	448 (2.76)	< 0.0001
Lipid-lowering drugs [n (%)]	757 (0.88)	559 (0.80)	198 (1.22)	< 0.0001
Proportions of relevant diseases or taking relevant medications during follow-up				
Myocardial infarction [n (%)]	1426 (1.65)	1016 (1.45)	410 (2.52)	< 0.0001
Hypertension [n (%)]	28,728 (33.31)	23,608 (33.72)	5120 (31.54)	< 0.0001
Diabetes [n (%)]	11,943 (13.85)	9336 (13.34)	2607 (16.06)	< 0.0001
Antihypertensive drugs [n (%)]	12,843 (14.89)	9640 (13.77)	3203 (19.73)	< 0.0001
Hypoglycemic drugs [n (%)]	4778 (5.54)	3285 (4.69)	1493 (9.19)	< 0.0001
Lipid-lowering drugs [n (%)]	2087 (2.42)	1530 (2.18)	557 (3.43)	< 0.0001

The measurement data of normal distribution were expressed as mean ± SD. The measurement data of skewed distribution were expressed as median (p25 ~ p75), and the counting data were expressed as number of cases (%). BMI, body mass index; DBP, diastolic blood pressure; eGFR, estimated glomerular filtration rate; FBG, fasting blood glucose; hsCRP, high sensitivity C reactive protein; SBP, systolic blood pressure; TC, cholesterol; UA, uric acid

### Effect of different hsCRP levels on the development of cardiac conduction disorders

**Fig. 2:**
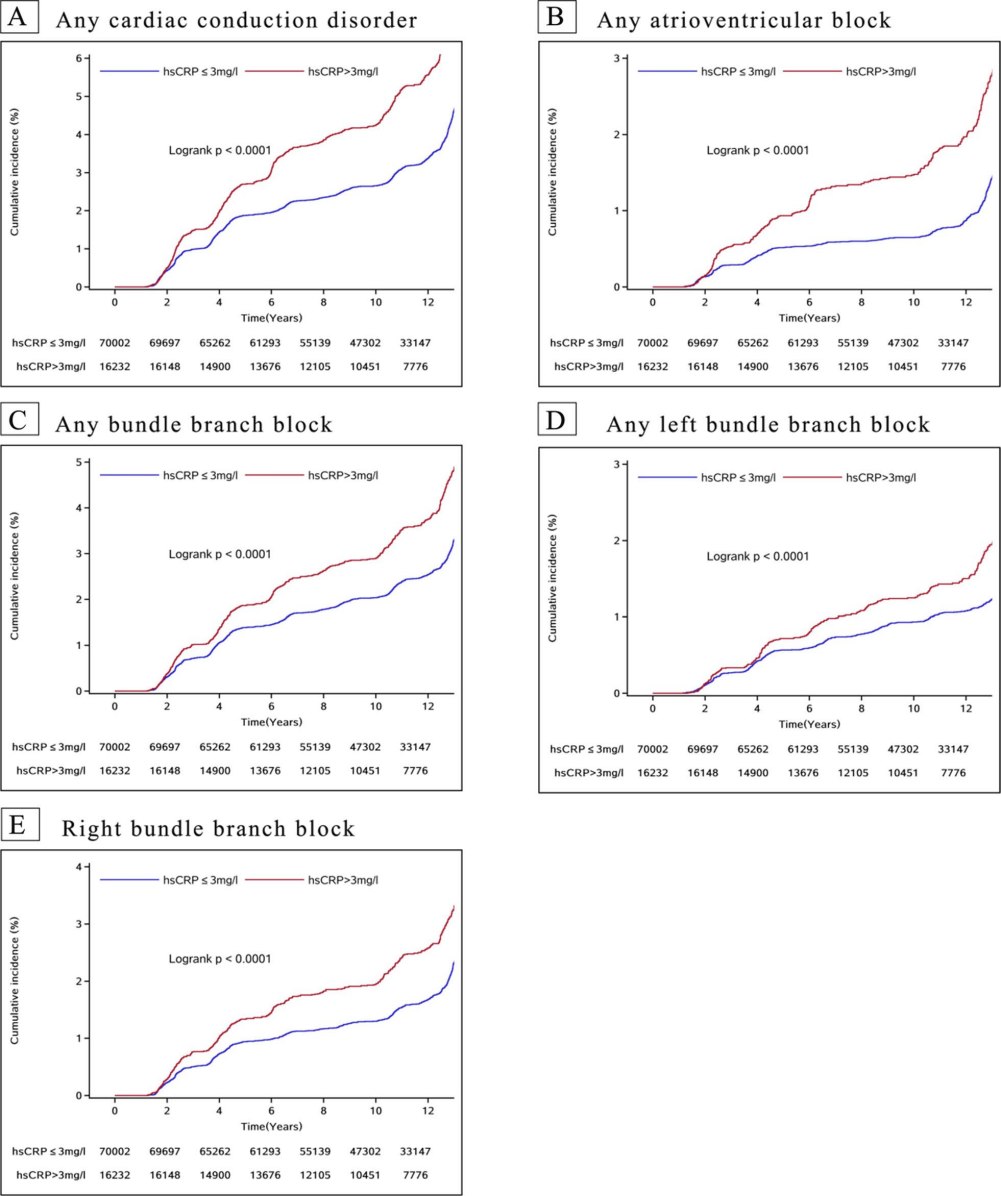
Cumulative incidence of the conduction disorders. **A**, Any cardiac conduction disorder; **B**, Any atrioventricular block; **C**, Any bundle branch block; **D**, Any left bundle branch block; **E**, Right bundle branch block

At a median follow-up of 11.83 (8.87–13.04) years, 3614 cases of heart conduction disorders occurred, and the incidence of conduction disorders in the total population was 3.96/1000 person-years. Figure 2 demonstrates the cumulative incidence of the groups. The log-rank test revealed a statistically significant difference in the cumulative incidence of the two groups (*P* < 0.0001).

With the occurrence of heart block as a dependent variable, and the different hsCRP groupings as the independent variable or each 1 standard deviation increase in loghsCRP as the independent variable, the age, sex, BMI, physical activity, smoking, alcohol consumption, TC, UA, eGFR at baseline, hypertension, diabetes, myocardial infarction at baseline or during follow-up and antihypertensive, lipid-lowering and glucose-lowering medication at baseline or during follow-up were corrected. Multifactorial Cox regression analysis revealed a 1.40-fold (95% CI 1.29–1.51) increased risk of developing aCCD, 1.64-fold (95% CI 1.44–1.87) increased risk of developing aAVB, and 1.29-fold (95% CI = 1.18–1.42) increased risk of developing aBBB in the hsCRP > 3 mg/L group compared with the hsCRP ≤ 3 mg/L group. Furthermore, a 1.25-fold (95% CI 1.07–1.45) increased risk of developing aLBBB and 1.31-fold (95% CI 1.17–1.47) increased risk of developing RBBB was observed. For each standard deviation increase in log (hsCRP), the risk of developing aCCD increased by 1.13-fold (95% CI 1.10–1.18), aAVB increased by 1.23-fold (95% CI 1.15–1.31), aBBB increased by 1.10-fold (95% CI 1.06–1.15), aLBBB increased by 1.04-fold (95% CI 0.97–1.11) and RBBB increased by 1.13-fold (95% CI 1.08–1.19) (Table 2).

**Table 2:** Multivariate Cox proportional hazard model for the incidence of conduction disorders

	Cases/Total	Incidence/1000-person years	Model 1 HR (95% CI)	Model 2 HR (95%CI)	Model 3 HR (95%CI)	Model 4 HR (95%CI)
Any Cardiac conduction disorder						
hsCRP ≤ 3 mg/L	2674/70002	3.59 (3.46–3.74)	Reference	Reference	Reference	Reference
hsCRP > 3 mg/L	940/16232	5.60 (5.25–5.97)	1.43 (1.33–1.54)	1.43 (1.32–1.54)	1.41 (1.31–1.53)	1.40 (1.29–1.51)
log (hsCRP)/SD	3614/86234	3.96 (3.83–4.09)	1.17 (1.13–1.21)	1.15 (1.11–1.19)	1.14 (1.11–1.19)	1.13 (1.10–1.18)
Any Atrioventricular Block						
hsCRP ≤ 3 mg/L	781/70002	1.05 (0.98–1.13)	Reference	Reference	Reference	Reference
hsCRP > 3 mg/L	340/16232	2.02 (1.82–2.25)	1.74 (1.53–1.98)	1.70 (1.49–1.94)	1.69 (1.48–1.93)	1.64 (1.44–1.87)
log (hsCRP)/SD	1121/86234	1.23 (1.16–1.30)	1.29 (1.22–1.37)	1.25 (1.18–1.33)	1.24 (1.17–1.33)	1.23 (1.15–1.31)
Any Bundle- branch Block						
hsCRP ≤ 3 mg/L	1921/70002	2.58 (2.47–2.70)	Reference	Reference	Reference	Reference
hsCRP > 3 mg/L	615/16232	3.66 (3.38–3.96)	1.31 (1.19–1.43)	1.32 (1.20–1.45)	1.31 (1.18–1.43)	1.29 (1.18–1.42)
log (hsCRP)/SD	2536/86234	2.78 (2.67–2.89)	1.12 (1.08–1.16)	1.11 (1.07–1.16)	1.11 (1.06–1.15)	1.10 (1.06–1.15)
Any Left Bundle- branch Block						
hsCRP ≤ 3 mg/L	744/70002	1.00 (0.93–1.07)	Reference	Reference	Reference	Reference
hsCRP > 3 mg/L	241/16232	1.43 (1.26–1.63)	1.30 (1.12–1.51)	1.26 (1.08–1.46)	1.26 (1.08–1.46)	1.25 (1.07–1.45)
log (hsCRP)/SD	985/86234	1.08 (1.02–1.15)	1.07 (1.01–1.14)	1.05 (0.98–1.12)	1.04 (0.98–1.11)	1.04 (0.97–1.11)
Right Bundle- branch Block						
hsCRP ≤ 3 mg/L	1320/70002	1.77 (1.68–1.87)	Reference	Reference	Reference	Reference
hsCRP > 3 mg/L	420/16232	2.50 (2.27–2.75)	1.31 (1.16–1.46)	1.35 (1.20–1.51)	1.33 (1.18–1.49)	1.31 (1.17–1.47)
log (hsCRP)/SD	1740/86234	1.90 (1.82–2.00)	1.15 (1.09–1.20)	1.15 (1.09–1.21)	1.14 (1.09–1.20)	1.13 (1.08–1.19)

Model 1: Corrected for age and gender

Model 2: Corrected for baseline body mass index, physical activity, smoking, alcohol consumption, cholesterol, uric acid, estimated glomerular filtration rate, hypertension, diabetes mellitus, and myocardial infarction based on model 1

Model 3: Corrected for new-onset hypertension, diabetes mellitus, and myocardial infarction during the follow-up based on model 2

Model 4: Corrected for those taking antihypertensive, lipid-lowering, and glucose-lowering drugs at baseline or during the follow-up based on model 3

HR, hazard ratio; Log (hsCRP)/SD, log-transformed for each standard deviation increase in hsCRP; CI, confidence interval

**Fig. 3:**
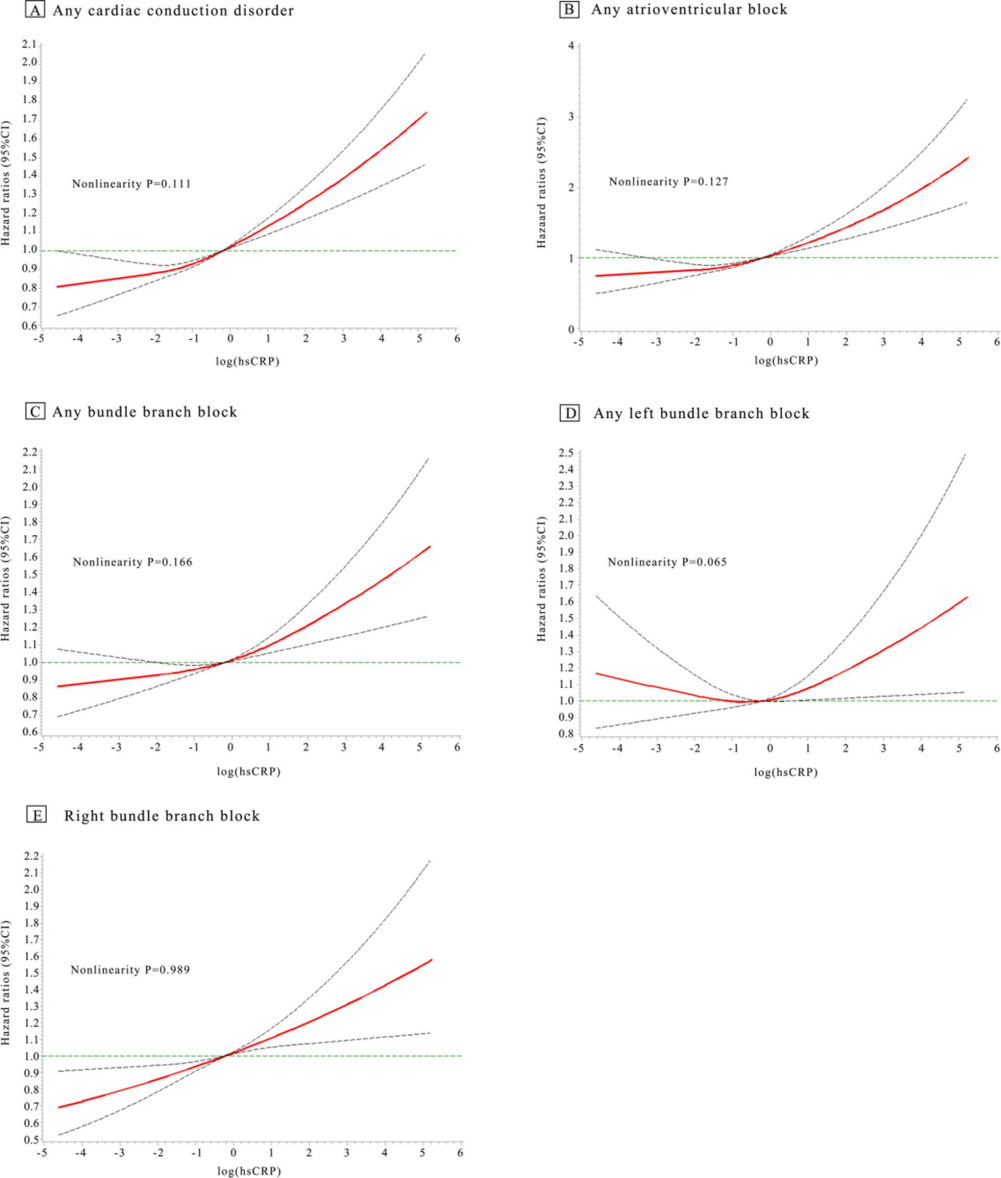
Restricted cubic spline curves of the effect of log (high-sensitivity C-reactive protein) on the onset of conduction disorders. **A**, Any cardiac conduction disorder; **B**, Any atrioventricular block; **C**, Any bundle branch block; **D**, Any left bundle branch block; **E**, Right bundle branch block

After correcting for the confounders mentioned above, a restricted spline curve was constructed by plotting log (hsCRP) against the risk of various cardiac conduction disorders. It revealed that log (hsCRP) was linearly correlated with the risk of aCCD, aAVB, aBBB, and RBBB (overall-association *P* < 0.0001, nonlinearity *P* > 0.05) (Fig. 3).

### Subgroup analysis

To identify the priority population, we conducted subgroup analyses based on sex and age. Following adjustment for the same confounding factors, the Cox regression results revealed that the risk of developing aCCD was elevated in both the male and non-elderly subgroups within the hsCRP > 3 mg/L category, when compared to the hsCRP ≤ 3 mg/L group. The HRs and their corresponding 95% CIs were 1.39 (1.28–1.51) and 1.44 (1.31–1.58), respectively (Table 3).

**Table 3:**
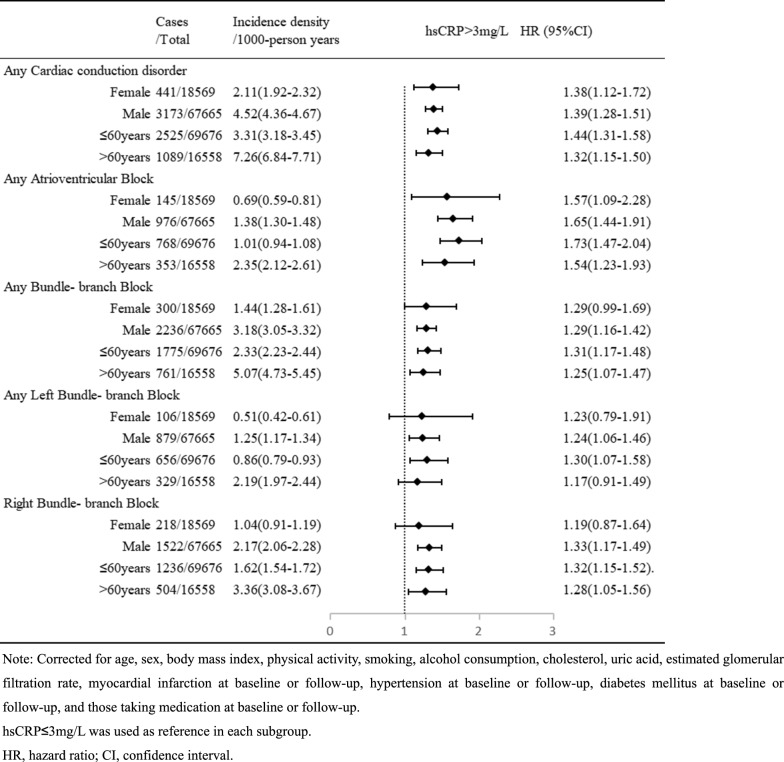
Subgroup analyses of the association between high-sensitivity C-reactive protein (hsCRP) and incidence of conduction disorders

### Sensitivity analysis

Guidelines recommend [24] a cutoff value of < 1 mg/L for low-level hsCRP. We performed Cox regression analysis by performing regrouping at this cutoff value and observed a 1.19-fold increased risk of developing aCCD in the hsCRP ≥ 1 mg/L group compared with the hsCRP < 1 mg/L group (95% CI 1.11–1.27) after correcting for the same confounding factors. To mitigate the impact of acute inflammatory responses and MI on the final event outcome, we conducted a subsequent Cox regression analysis. This analysis involved the exclusion of individuals with baseline hsCRP > 10 mg/L and those with a history of MI, respectively. In our investigation, we observed a 1.41-fold increase in risk (95% CI 1.30–1.53) and 1.39-fold increase in risk (95% CI 1.29–1.5), respectively, for the development of aCCD condition among individuals with hsCRP > 3 mg/L when compared to those with hsCRP ≤ 3 mg/L. Additionally, for each increase in log (hsCRP), corresponding to an increase of 1 standard deviation, we identified a 1.14-fold elevated risk (95% CI 1.10–1.19) and 1.14-fold increased risk (95% CI 1.10–1.18), respectively, for the development of aCCD. These findings are in alignment with those obtained from the overall study population (Table 4).

**Table 4:** Sensitivity analyses of the association between high-sensitivity C-reactive protein (hsCRP) and incidence of conduction disorders

	Cases/Total	Incidence/1000-person years	Model 1 HR (95% CI)	Model 2 HR (95%CI)	Model 3 HR (95%CI)	Model 4 HR (95%CI)
Regrouping of hsCRP						
Any Cardiac conduction disorder						
hsCRP < 1 mg/L	1800/48180	3.48 (3.33–3.65)	Reference	Reference	Reference	Reference
hsCRP ≥ 1 mg/L	1814/38054	4.58 (4.38–4.81)	1.26 (1.18–1.34)	1.22 (1.14–1.30)	1.21 (1.13–1.29)	1.19 (1.11–1.27)
Any Atrioventricular Block						
hsCRP < 1 mg/L	511/48180	0.99 (0.91–1.08)	Reference	Reference	Reference	Reference
hsCRP ≥ 1 mg/L	610/38054	1.54 (1.43–1.67)	1.49 (1.32–1.67)	1.38 (1.22–1.56)	1.37 (1.21–1.54)	1.33 (1.17–1.50)
Any Bundle- branch Block						
hsCRP < 1 mg/L	1307/48180	2.53 (2.39–2.67)	Reference	Reference	Reference	Reference
hsCRP ≥ 1 mg/L	1229/38054	3.11 (2.94–3.29)	1.17 (1.08–1.27)	1.15 (1.06–1.25)	1.14 (1.05–1.24)	1.13 (1.05–1.23)
Any Left Bundle- branch Block						
hsCRP < 1 mg/L	523/48180	1.01 (0.93–1.10)	Reference	Reference	Reference	Reference
hsCRP ≥ 1 mg/L	462/38054	1.17 (1.07–1.28)	1.08 (0.95–1.22)	1.03 (0.91–1.17)	1.03 (0.90–1.17)	1.02 (0.90–1.17)
Right Bundle- branch Block						
hsCRP < 1 mg/L	877/48180	1.69 (1.59–1.81)	Reference	Reference	Reference	Reference
hsCRP ≥ 1 mg/L	863/38054	2.18 (2.04–2.33)	1.24 (1.12–1.36)	1.24 (1.12–1.36)	1.22 (1.11–1.34)	1.21 (1.09–1.33)
Excluding hsCRP > 10 mg/L at baseline (N = 3342)						
Any Cardiac conduction disorder						
hsCRP ≤ 3 mg/L	2674/70002	3.59 (3.46–3.74)	Reference	Reference	Reference	Reference
hsCRP > 3 mg/L	758/12890	5.66 (5.27–6.08)	1.44 (1.33–1.57)	1.44 (1.33–1.56)	1.43 (1.32–1.55)	1.41 (1.30–1.53)
log (hsCRP)/SD	3432/82892	3.91 (3.78–4.04)	1.18 (1.14–1.23)	1.16 (1.12–1.21)	1.15 (1.11–1.20)	1.14 (1.10–1.19)
Any Atrioventricular Block						
hsCRP ≤ 3 mg/L	781/70002	1.05 (0.98–1.13)	Reference	Reference	Reference	Reference
hsCRP > 3 mg/L	274/12890	2.05 (1.82–2.30)	1.75 (1.52–2.02)	1.71 (1.49–1.97)	1.70 (1.47–1.96)	1.66 (1.44–1.92)
log (hsCRP)/SD	1055/82892	1.20 (1.13–1.28)	1.32 (1.23–1.41)	1.27 (1.18–1.36)	1.26 (1.17–1.36)	1.25 (1.16–1.34)
Any Bundle- branch Block						
hsCRP ≤ 3 mg/L	1921/70002	2.58 (2.47–2.70)	Reference	Reference	Reference	Reference
hsCRP > 3 mg/L	495/12890	3.69 (3.38–4.04)	1.32 (1.19–1.46)	1.33 (1.20–1.47)	1.32 (1.19–1.46)	1.31 (1.18–1.45)
log (hsCRP)/SD	2416/82892	2.75 (2.64–2.86)	1.13 (1.08–1.18)	1.12 (1.07–1.17)	1.11 (1.06–1.16)	1.10 (1.05–1.15)
Any Left Bundle- branch Block						
hsCRP ≤ 3 mg/L	744/70002	1.00 (0.93–1.07)	Reference	Reference	Reference	Reference
hsCRP > 3 mg/L	194/12890	1.45 (1.26–1.67)	1.32 (1.12–1.55)	1.28 (1.09–1.51)	1.28 (1.09–1.51)	1.27 (1.08–1.50)
log (hsCRP)/SD	938/82892	1.07 (1.00–1.14)	1.08 (1.00–1.16)	1.05 (0.97–1.12)	1.04 (0.97–1.12)	1.04 (0.97–1.12)
Right Bundle- branch Block						
hsCRP ≤ 3 mg/L	1320/70002	1.77 (1.68–1.87)	Reference	Reference	Reference	Reference
hsCRP > 3 mg/L	339/12890	2.53 (2.27–2.81)	1.32 (1.17–1.48)	1.36 (1.20–1.54)	1.34 (1.19–1.52)	1.33 (1.17–1.50)
log (hsCRP)/SD	1659/82892	1.89 (1.80–1.98)	1.16 (1.10–1.23)	1.16 (1.10–1.23)	1.15 (1.09–1.22)	1.14 (1.08–1.21)
Excluding Myocardial Infarction at baseline (N = 965)						
Any Cardiac conduction disorder						
hsCRP ≤ 3 mg/L	2629/69270	3.56 (3.43–3.70)	Reference	Reference	Reference	Reference
hsCRP > 3 mg/L	919/15999	5.54 (5.19–5.19)	1.43 (1.32–1.54)	1.43 (1.32–1.54)	1.42 (1.31–1.53)	1.39 (1.29–1.51)
log (hsCRP)/SD	3548/85269	3.93 (3.80–4.06)	1.17 (1.13–1.21)	1.15 (1.11–1.19)	1.15 (1.11–1.18)	1.14 (1.10–1.18)
Any Atrioventricular Block						
hsCRP ≤ 3 mg/L	770/69270	1.04 (0.97–1.12)	Reference	Reference	Reference	Reference
hsCRP > 3 mg/L	332/15999	2.00 (1.79–2.23)	1.73 (1.52–1.97)	1.69 (1.48–1.93)	1.68 (1.47–1.92)	1.63 (1.43–1.87)
log (hsCRP)/SD	1102/85269	1.22 (1.15–1.29)	1.28 (1.21–1.37)	1.25 (1.17–1.33)	1.24 (1.17–1.32)	1.22 (1.15–1.30)
Any Bundle- branch Block						
hsCRP ≤ 3 mg/L	1887/69270	2.56 (2.45–2.68)	Reference	Reference	Reference	Reference
hsCRP > 3 mg/L	601/15999	3.62 (3.34–3.92)	1.31 (1.18–1.43)	1.32 (1.20–1.45)	1.31 (1.19–1.44)	1.30 (1.18–1.43)
log (hsCRP)/SD	2488/85269	2.75 (2.65–2.86)	1.12 (1.01–1.17)	1.11 (1.07–1.16)	1.11 (1.06–1.15)	1.10 (1.06–1.15)
Any Left Bundle- branch Block						
hsCRP ≤ 3 mg/L	734/69270	0.99 (0.93–1.07)	Reference	Reference	Reference	Reference
hsCRP > 3 mg/L	231/15999	1.39 (1.23–1.58)	1.27 (1.09–1.48)	1.23 (1.05–1.43)	1.23 (1.06–1.44)	1.22 (1.05–1.43)
log (hsCRP)/SD	965/85269	1.97 (1.00–1.14)	1.07 (1.00–1.14)	1.04 (0.97–1.11)	1.04 (0.97–1.11)	1.03 (0.96–1.11)
Right Bundle- branch Block						
hsCRP ≤ 3 mg/L	1295/69270	1.75 (1.66–1.86)	Reference	Reference	Reference	Reference
hsCRP > 3 mg/L	414/15999	2.49 (2.27–2.75)	1.31 (1.17–1.46)	1.36 (1.21–1.52)	1.34 (1.19–1.51)	1.33 (1.18–1.49)
log (hsCRP)/SD	1709/85269	1.89 (1.81–1.98)	1.15 (1.09–1.21)	1.15 (1.10–1.21)	1.15 (1.09–1.20)	1.14 (1.01–1.20)

Model 1: Corrected for age and gender

Model 2: Corrected for baseline body mass index, physical activity, smoking, alcohol consumption, cholesterol, uric acid, estimated glomerular filtration rate, hypertension, diabetes mellitus, and myocardial infarction based on model 1

Model 3: Corrected for new-onset hypertension, diabetes mellitus, and myocardial infarction during the follow-up based on model 2

Model 4: Corrected for those taking antihypertensive, lipid-lowering, and glucose-lowering drugs at baseline or during the follow-up based on model 3

HR, hazard ratio; Log (hsCRP)/SD, log-transformed for each standard deviation increase in hsCRP; CI, confidence interval

The entire follow-up period of the present study was long. As follow-up was performed every 2 years, we divided the entire follow-up interval into six segments using a 2-year interval. A time-dependent Cox regression model was used to analyze the short-term exposure effect of increased hsCRP levels on the risk of developing cardiac conduction disorder. After correcting for sex and time-varying risk factors such as age, BMI, smoking, alcohol consumption, physical activity, TC, UA, eGFR, hypertension, diabetes mellitus, myocardial infarction, and taking antihypertensive, lipid-lowering, and glucose-lowering drugs, we observed that compared with the hsCRP ≤ 3 mg/L group, the risk of developing aCCD increased 1.16-fold in the hsCRP > 3 mg/L group (95% CI 1.11–1.21). Furthermore, for every 1 standard deviation increase in log (hsCRP), the risk of developing aCCD increased by 1.04-fold (95% CI 1.02–1.05) (Additional file 1: Table S2).

Overall, 1632 all-cause deaths (1.89%) were reported in the present study. To eliminate the effect of all-cause mortality events on the outcomes during follow-up, we performed a competing risk of death model analysis on the total population; the results were consistent with those of the total population after correcting for the same confounders mentioned above (Additional file 1: Table S3).

## Discussion

In the present study, we confirmed that elevated hsCRP levels increase the risk of developing various cardiac conduction disorders and that hsCRP levels are associated with a dose–response risk of developing conduction disorders. This increased risk is independent of the traditional risk factors but age- and sex-dependent.

We identified high hsCRP levels as a risk factor for cardiac conduction disorders, with an increased risk of developing both atrioventricular block and right and left bundle branch block compared with the hsCRP ≤ 3 mg/L group. Moreover, the strength of the association between hsCRP levels and atrioventricular block was greater than that between hsCRP and bundle branch block. To the best of our knowledge, this is the first large-scale prospective cohort study to identify an association between hsCRP and cardiac conduction disorders. Only Emilie et al. [16] reported a 1.07-fold increased risk of developing cardiac conduction disorders for every 10 mg/L increase in hsCRP levels in a cohort of 4314 older adults. Although there are no previous studies similar to ours, the association between high hsCRP and chronic noncommunicable diseases, including atherosclerotic cardiovascular disease, has been consistently confirmed [25–28]. Our findings expand the existing knowledge on chronic inflammation and the risk of developing cardiovascular diseases, as indicated by hsCRP levels.

We not only identified high hsCRP level as a risk factor for cardiac conduction disorders but also demonstrated a dose–response association between the inflammatory marker hsCRP and the risk of cardiac conduction disorders. For every 1 standard deviation increase in log (hsCRP), the risk of cardiac block, atrioventricular block, and right bundle branch block increased. Furthermore, the restricted spline function confirmed a linear association between log (hsCRP) and cardiac conduction block and atrioventricular block and right bundle branch block. The UK Biobank study [29] reported a dose-dependent association between hsCRP and the risk of bradyarrhythmia, with a 1.15-fold increased risk of bradyarrhythmia in the (3.0, 4.0) mg/L group, a 1.18-fold increased risk in the (4.0, 10.0) mg/L group, and a 1.30-fold increased risk in the ≥ 10.0 mg/L group compared with the hsCRP < 0.5 mg/L group. Moreover, a meta-analysis confirmed a linear association between hsCRP and the risk of cardiovascular diseases, stroke, and coronary heart disease [30]. Taken together, these studies indicate a dose–response relationship between inflammation levels and adverse cardiovascular outcomes, including conduction disorders. We should attempt to decrease the risk of cardiovascular diseases, including conduction disorders, by reducing inflammation levels in the body by embracing a healthy lifestyle, including a healthy diet, exercise, smoking cessation, and weight loss [31, 32].

Furthermore, we noted that elevated hsCRP levels contribute to an augmented risk of cardiac conduction abnormalities. This risk manifests in a manner influenced by sex and age, with a more pronounced increase in cardiac conduction disorder risk observed in the male and younger age subgroups. Many studies have confirmed that the male sex is a risk factor for cardiac conduction disorders development [9–11, 33]. Data from Asian countries have revealed that male sex is associated with increased hsCRP levels [34, 35]; as a result, there may be a combined or superimposed effect of the inflammatory response among men for the development of cardiac conduction disorders, resulting in a more pronounced increased risk in this population. Previous studies have described a correlation between advanced age and increased risk of developing cardiac conduction disorders [10, 11]. However, in the present study, we observed that the pathogenic risk of high hsCRP level was higher in the younger subgroup. Studies have demonstrated that elevated hsCRP levels may be associated with traditional cardiovascular risk factors such as blood pressure, blood glucose levels, and obesity [36, 37]; therefore, the possibility of combined cardiovascular risk factors is high in the younger population with elevated hsCRP levels. In addition, studies have shown that the risk of developing cardiovascular diseases is associated with the age of onset of diabetes or hypertension; the younger the age of onset, the higher the relative risk of developing cardiovascular diseases [38, 39]. These findings may explain the higher risk of conduction disorders in the younger population than in the older population when they are simultaneously exposed to high hsCRP levels.

To determine the robustness of the study results, we performed Cox regression analysis via regrouping with or without hsCRP < 1 mg/L in sensitivity analysis. We observed that the risk of developing any cardiac conduction disorder increased 1.19-fold (95% CI 1.11–1.27) in the hsCRP ≥ 1 mg/L group compared with the hsCRP < 1 mg/L group, suggesting that even if individual hsCRP levels decreased to 1 mg/L, the risk of conduction disorder still increased. We conducted a repeated Cox regression analysis, excluding individuals with baseline hsCRP > 10 mg/L or those with a history of MI. The outcomes of this analysis remained in accordance with the primary analysis. This suggests that the onset of conductive disorders owing to increased hsCRP levels was independent of whether the patient was complicated with an acute infection. We performed time-dependent Cox regression analysis over a 2-year time period and observed a 1.16-fold (95% CI 1.11–1.21) increased risk of developing any cardiac conduction disorder in the hsCRP > 3 mg/L group compared with the hsCRP ≤ 3 mg/L group, suggesting that decreasing the exposure to high hsCRP levels to 2 years could still increase the risk of developing conduction disorders. We considered the longer follow-up period and the possibility of competing risks of death; therefore, we conducted competing risk of death model analyses in the overall population; the results were consistent with those of the main analysis. Taken together, these sensitivity analyses confirm the robustness of our results.

A previous investigation proposed that CRP functions not only as an indicator of inflammation but also as a promoter of inflammation, thereby leading to myocardial fibrosis through the TLR4/NF-κB/TGF-β pathway [40]. Another study revealed that CRP assumes a proarrhythmic role by directly influencing calcium homeostasis in cases of cardiac conduction disorders [41]. In our current research, we have provided evidence that elevated hsCRP levels can increase the risk of conduction disorders. Importantly, this elevated risk persists even after accounting for conventional cardiovascular risk factors such as hypertension, diabetes mellitus, and myocardial infarction. Therefore, we suggest that the aforementioned inflammatory responses may directly contribute to the development of conduction disorders. Our subgroup and sensitivity analyses yielded results consistent with those observed in the general population. These findings validate previous research and confirm the pivotal role played by CRP in the pathogenesis of these conditions. However, it is crucial to acknowledge that potential comorbidities and lifestyle habits associated with elevated hsCRP levels may also contribute to the onset of cardiac conduction disorders. These factors should be taken into consideration in the prevention of conduction-related diseases.

Several limitations of the present study has to be addressed. First, cardiac conduction disorders were diagnosed based on the ECG measurements taken at the medical check-up and did not include patients with conduction disorders who missed the follow-ups, which may have decreased the efficacy of the analysis. Second, the study population was employees of the Kailuan group, a predominantly male population, with limited extrapolation of the results; however, subgroup analysis of sex was conducted to compensate for this limitation. Third, hsCRP levels and other baseline assessments were only measured once; therefore, the results are not a representation of the long-term levels of the study population. Nevertheless, we performed time-dependent Cox regression analysis by considering changes in hsCRP and other baseline variables during the follow-up period. The results were still statistically different, reducing the effect of the length of follow-up. Fourthly, it is essential to note that this study is observational in nature. Consequently, it cannot establish a definitive causal link between systemic inflammation and cardiac conduction disorders. To validate such a relationship, further confirmation through randomized controlled studies is warranted.

## Conclusions

The current study illustrates a significant connection between hsCRP levels and the risk of cardiac conduction disorders. These findings shed light on a potential new avenue for the prevention and management of cardiac conduction disorders.

### Supplementary Information

**Additional file 1: Table S1.** Diagnostic criteria for the different conduction disorders. **Table S2.** Association between time-dependent high-sensitivity C-reactive protein (hsCRP) and risk of outcomes. **Table S3.** Competing risk model of death for the association between high-sensitivity C-reactive protein (hsCRP) and incidence of conduction disorders.

## Data Availability

The data underlying this article will be shared on reasonable request to the corresponding authors.
