# Supplementary material for: Elevated high-sensitivity C-reactive protein levels increase the risk of new-onset cardiac conduction disorders
Source: Cardiovasc Diabetol. 2023 Sep 30;22:268. doi: 10.1186/s12933-023-01987-1 (PMC10543876; doi:10.1186/s12933-023-01987-1)
Supplement: Supplementary file 1 — Additional file 1: Table S1. Diagnostic criteria for the different conduction disorders. Table S2. Association between time-dependent high-sensitivity C-reactive protein (hsCRP) and risk of outcomes. Table S3. Competing risk model of death for the association between high-sensitivity C-reactive protein (hsCRP) and incidence of conduction disorders. [file 12933_2023_1987_MOESM1_ESM.docx]

Additional file 1: Table S1. Diagnostic criteria for the different conduction disorders.

| Cardiac conduction disorder | Minnesota code | Diagnostic criteria |
| --- | --- | --- |
| First-degree Atrioventricular Block (I°AVB) | 6-3 | 1. PR interval ≥200ms; 2. Each atrial excitement is transmitted to the ventricle. |
| Second-degree Atrioventricular Block (II°AVB) | 6-2 | II°AVB is subclassified as type I and type II.   1. Type I is characterized by progressive prolongation of the PR interval before a non-conducted beat and a shorter PR interval after the blocked beat and is usually observed together with QRS； 2. Type II is characterized by fixed PR intervals before and after blocked beats and is usually associated with a wide QRS complex. |
| Third-degree Atrioventricular Block (III°AVB) | 6-1 | Complete absence of atrioventricular conduction. |
| Complete right bundle branch block (CRBBB) | 7-2 | 1. QRS duration ≥120 ms; 2. rsr’, rsR’or rSR’ in leads V1 or V2. The R’ or r’ deflection is usually wider than that of the initial r wave.In a minority of patients, a wide and often notched R wave pattern (absence of an s/S wave) may be observed in lead V1 and/or V2; 3. S wave of greater duration than that of the R wave or ≥40ms in leads I and V6. |
| Incomplete right bundle branch block (iRBBB) | 7-3 | 1. QRS duration: 110 -119 ms; 2. Other criteria are the same as those for CRBBB. |
| Complete left bundle branch block  (CLBBB) | 7-1 | 1. QRS duration ≥120 ms; 2. Broad notched or slurred R wave in leads I , aVL , V5 and V6; 3. Absence of q waves in leads I, V5 and V6; 4. R peak time of ≥60 ms in leads V5 and V6. |
| Incomplete Left bundle branch block (iLBBB) | 7-6 | 1. QRS duration: 110 -119 ms; 2. Presence of a left ventricular hypertrophy pattern; 3. R peak time of ≥60 ms in leads V4, V5, and V6; 4. Absence of the q wave in leads I, V5, and V6. |
| Left anterior fascicular block (LAFB) |  | 1. QRS duration <120ms; 2. Frontal plane axis between −45° and −90°; 3. R-peak time in lead aVL of ≥45 ms; 4. qR patternin in lead aVL; 5. rS patternin in leads II, III, and avF. |
| Left posterior fascicular block (LPFB) |  | 1. QRS duration <120ms; 2. Frontal plane axis between 90° and 180°; 3. rS pattern in leads I and aVL; 4. qR pattern in leads III and aVF. |
| Nonspecific intraventricular conduction disturbance (NS-IVCD) | 7-4 | QRS duration >110 ms when both RBBB and LBBB are excluded. |

Additional file 1: Table S2. Association between time-dependent high-sensitivity C-reactive protein (hsCRP) and risk of outcomes.

|  | Model 1  HR(95% CI) | Model 2  HR (95%CI) | Model 3  HR(95%CI) |
| --- | --- | --- | --- |
| Any Cardiac conduction disorder |  |  |  |
| hsCRP≤3mg/L | Reference | Reference | Reference |
| hsCRP>3mg/L | 1.20(1.15-1.25) | 1.17(1.12-1.22) | 1.16(1.11-1.21) |
| log(hsCRP)/SD | 1.06(1.04-1.07) | 1.04(1.02-1.06) | 1.04(1.02-1.05) |
| Any Atrioventricular Block |  |  |  |
| hsCRP≤3mg/L | Reference | Reference | Reference |
| hsCRP>3mg/L | 1.26(1.17-1.35) | 1.20(1.12-1.29) | 1.19(1.11-1.28) |
| log(hsCRP)/SD | 1.09(1.05-1.12) | 1.06(1.03-1.09) | 1.05(1.02-1.09) |
| Any Bundle- branch Block |  |  |  |
| hsCRP≤3mg/L | Reference | Reference | Reference |
| hsCRP>3mg/L | 1.18(1.12-1.24) | 1.16(1.09-1.22) | 1.15(1.10-1.22) |
| log(hsCRP)/SD | 1.04(1.02-1.07) | 1.03(1.01-1.05) | 1.03(1.01-1.05) |
| Any Left Bundle- branch Block |  |  |  |
| hsCRP≤3mg/L | Reference | Reference | Reference |
| hsCRP>3mg/L | 1.28(1.18-1.38) | 1.22(1.12-1.32) | 1.22(1.12-1.32) |
| log(hsCRP)/SD | 1.06(1.02-1.09) | 1.03(0.99-1.06) | 1.03(0.99-1.06) |
| Right Bundle- branch Block |  |  |  |
| hsCRP≤3mg/L | Reference | Reference | Reference |
| hsCRP>3mg/L | 1.15(1.09-1.22) | 1.15(1.08-1.22) | 1.14(1.08-1.22) |
| log(hsCRP)/SD | 1.04(1.01-1.07) | 1.03(1.01-1.06) | 1.03(1.01-1.06) |

Note：Model 1: Corrected for sex and age;

Model 2: Corrected for body mass index, smoking, alcohol consumption, physical activity, hypertension, diabetes mellitus, myocardial infarction, cholesterol, uric acid, and estimated glomerular filtration rate based on model 1;

Model 3: Corrected for taking antihypertensive, lipid-lowering, and glucose-lowering drugs based model 2.

HR, hazard ratio; Log(hsCRP)/SD, log-transformed for each standard deviation increase in hsCRP; CI, confidence interval.

Additional file 1: Table S3. Competing risk model of death for the association between high-sensitivity C-reactive protein (hsCRP) and incidence of conduction disorders.

|  | Model 1  HR(95% CI) | Model 2  HR (95%CI) | Model 3  HR(95%CI) | Model 4  HR(95%CI) |
| --- | --- | --- | --- | --- |
| Any Cardiac conduction disorder |  |  |  |  |
| hsCRP≤3mg/L | Reference | Reference | Reference | Reference |
| hsCRP>3mg/L | 1.42(1.31-1.53) | 1.41(1.31-1.53) | 1.40(1.30-1.51) | 1.38(1.28-1.49) |
| log(hsCRP)/SD | 1.16(1.12-1.20) | 1.15(1.11-1.19) | 1.14(1.10-1.18) | 1.13(1.09-1.17) |
| Any Atrioventricular Block |  |  |  |  |
| hsCRP≤3mg/L | Reference | Reference | Reference | Reference |
| hsCRP>3mg/L | 1.72(1.51-1.96) | 1.68(1.48-1.92) | 1.67(1.47-1.90) | 1.62(1.43-1.85) |
| log(hsCRP)/SD | 1.28(1.21-1.37) | 1.25(1.17-1.33) | 1.24(1.16-1.32) | 1.22(1.15-1.30) |
| Any Bundle- branch Block |  |  |  |  |
| hsCRP≤3mg/L | Reference | Reference | Reference | Reference |
| hsCRP>3mg/L | 1.29(1.18-1.42) | 1.31(1.19-1.43) | 1.29(1.18-1.42) | 1.28(1.17-1.41) |
| log(hsCRP)/SD | 1.11(1.07-1.16) | 1.11(1.06-1.15) | 1.10(1.05-1.15) | 1.09(1.05-1.14) |
| Any Left Bundle- branch Block |  |  |  |  |
| hsCRP≤3mg/L | Reference | Reference | Reference | Reference |
| hsCRP>3mg/L | 1.28(1.11-1.49) | 1.25(1.07-1.48) | 1.24(1.07-1.44) | 1.23(1.06-1.44) |
| log(hsCRP)/SD | 1.07(1.00-1.14) | 1.04(0.97-1.11) | 1.03(0.97-1.11) | 1.03(0.96-1.10) |
| Right Bundle- branch Block |  |  |  |  |
| hsCRP≤3mg/L | Reference | Reference | Reference | Reference |
| hsCRP>3mg/L | 1.29(1.15-1.44) | 1.33(1.19-1.49) | 1.31(1.17-1.47) | 1.30(1.16-1.46) |
| log(hsCRP)/SD | 1.14(1.09-1.19) | 1.14(1.09-1.21) | 1.13(1.08-1.19) | 1.13(1.07-1.18) |

Note: Model 1: Corrected for age and gender;

Model 2: Corrected for baseline body mass index, physical activity, smoking, alcohol consumption, cholesterol, uric acid, estimated glomerular filtration rate, hypertension, diabetes mellitus, and myocardial infarction based on model 1;

Model 3: Corrected for new-onset hypertension, diabetes mellitus, and myocardial infarction during the follow-up based on model 2;

Model 4: Corrected for those taking antihypertensive, lipid-lowering, and glucose-lowering drugs at baseline or during the follow-up based on model 3.

Log(hsCRP)/SD, log-transformed for each standard deviation increase in hsCRP.
